# The association between paternal prostate cancer and type 2 diabetes

**DOI:** 10.1186/1477-3163-6-14

**Published:** 2007-09-26

**Authors:** Peter Meyer, Christine Zuern, Norbert Hermanns, Thomas Haak

**Affiliations:** 1 Institute of Molecular Medicine, Munich, Germany; 2 Institute of Human Genetics, Division for Molecular Oncogenetics, University Hospital Tuebingen, Germany; 3 Research Institute of the Diabetes Academy, Bad Mergentheim, Germany

## Abstract

**Objective:**

Increasing evidence indicates that type 2 diabetic patients are at elevated risk for developing different kinds of cancers. However, diabetes mellitus may be a protective factor for prostate cancer since both were found to be negatively associated. Based on the same genetic background, parents of diabetic patients might show similar risks concerning cancers.

**Research design and methods:**

We conducted a case-control study, where familiy history of 794 type 2 diabetic cases and 775 non-diabetic controls was ascertained. Then, we expanded our study up to 801 type 2 diabetic cases and 1267 non-diabetic controls.

**Results:**

Concerning the 794 type 2 diabetic patients and 775 controls, we observed that cancer of cervix uteri was elevated among mothers of controls (odds ratio (OR) 0.19; 95% confidence interval (CI) 0.02 to 0.88; p = 0.033). Mothers of diabetic patients showed an increased history of cancers of the liver and biliary tract (OR 5.23; 95% CI 1.87 to 19.9; p = 0.0009) and stomach (OR 3.84; 95% CI 1.47 to 12.4; p = 0.0049). Pancreatic cancers were found to be elevated in fathers of diabetic patients (OR 4.92; 95% CI 1.07 to 46.7; p = 0.039). Most notably, a lower number of prostate cancers was observed in fathers of diabetic patients (OR 0.47; 95% CI 0.22 to 0.94; p = 0.032). Since diabetic patients were 14.3 years older than the controls, higher levels of cancer history among parents of diabetic patients would have been expected. Thus, the observed lower level of history of prostate cancer can be regarded as highly reliable.

The analysis of 801 type 2 diabetics and 1267 controls showed that cancer of stomach was elevated among mothers of controls (OR 2.67; p = 0.0106). In addition, stomach cancers were found to be elevated in fathers of diabetic patients (OR 2.10; p = 0.0141). In accordance with the previous investigation, we again obseved a lower number of prostate cancers in fathers of diabetic patients (OR 0.49; p = 0.0279).

However, the application of the statistical method of Mantel-Haenszel showed no significant result concerning any of the cancer histories.

**Conclusion:**

Fathers of patients suffering from type 2 diabetes were diagnosed less frequently with prostate cancer compared to fathers of non-diabetic controls. As first-degree relatives, e.g. diabetic patients and their fathers, share 50% of their genes, it appears plausible that genetic factors may play an important role in the negative association between diabetes and prostate cancer. However, different statistic analyses showed controversial results concerning the effect of type 2 diabetes on prostate cancers.

## Introduction

Diabetes has been associated with an increased risk of several human malignancies, including cancers of the pancreas [1,2], colon [3], endometrium [4,5], breast [6], kidney [7], liver [5,8], biliary tract [7] and esophagus [9].

However, a protective effect of diabetes mellitus on the risk of prostate cancer has been suggested by several [4,7]. but not all studies [10,11] It has also been reported that only long-term diabetes has a protective effect on prostate cancer [12,13].

Recently a large, population-based cohort study in Sweden found a significantly decreased risk of prostate cancer among men hospitalized for diabetes mellitus [14]. Further evidence comes from Rosenberg et al. who reported that white type 2 diabetic patients appear to have a lower risk of prostate cancer [15]. However, a two hospital-based case-control study conducted in Italy and Greece could not confirm these findings [13].

The objective of our study was to analyze the relationship of a diabetic genetic background and cancer. In this report, we present results from a case-control study conducted in Germany, where we ascertained the family history of cancers in type 2 diabetic patients and controls. In our investigations, we evaluated whether paternal history of prostate cancer was decreased among type 2 diabetic patients.

## Materials and methods

From September 2001 to February 2002, cases and controls were recruited in the southern part of Germany. The first case group consisted of 794 patients (median age 62.3 years), the second of 801 patients, who were hospitalised with a primary or secondary diagnosis of type 2 diabetes according to the International Classification of Diseases (ICD-10) in the Diabetes Hospital Bad Mergentheim, Germany. 775 control subjects (median age 47.9 years) and 1267 controls respectively were selected from the Center of blood donation Tuebingen, Germany. It was required that the controls never had been diagnosed with type 2 diabetes. This was verified by the measurement of HbA1c, the regular check on blood glucose by the Center of blood donation and a negative history of diabetes in the interview.

A complete interview was obtained from all cases and controls by two specifically trained interviewers. The interviews were based on structured questionnaires which included familiy history of differend kinds of cancers, e.g. cancers of the prostate, breast, colon and rectum, uterus, kidney, pancreas, esophagus, liver and biliary tract. All paternal and maternal types of cancer were considered in the statistical analysis, except data of participants being uncertain about their parental cancer history. Since we concentrated on the relationship of paternal prostate cancer and type 2 diabetes, the other cancers, which were found to be statistically significant, must be regarded as descriptive results. Therefore, an adjustment of p-values for multiple testing was not done. Odds ratios, 95% confidence intervals and p-values were calculated using the statistics package JMP Version 5. In order to obtain estimates of odds ratios also in tables with zero counts, we added 0.5 to all table entries. Based on significantly different median ages of patients and controls, terziles were built and the effect of age on the numbers of cancers was tested. We found that age had no influence on the numbers of cancers and is no biasing effect on the results.

## Results

The prevalence of history of paternal and maternal cancers and the correponding p-values, which were generated with 794 patients and 775 controls, are shown in Table 1. Statistically significant differences in cancer histories were observed in both fathers and mothers of diabetic patients and controls. These differences were found in cancers of cervix uteri, liver and biliary tract, stomach, pancreas and prostate.

**Table 1:** Frequencies of cancers in parents of 794 diabetic patients and 775 controls

	**Fathers of**				**Mothers of**			
**Type of cancer**	**diabetic patients**	**controls**			**diabetic patients**	**controls**		

	**%**	**%**	**p**	**OR**	**%**	**%**	**p**	**OR**

Basalioma	0.00	0.00	0.99	0.98	0.13	0.00	0.48	2.92
Brain & other nervous system	0.63	0.39	0.53	1.54	0.25	0.26	0.97	0.97
Breast	0.00	0.00	0.99	0.98	3.65	4.15	0.61	0.88
Cervix uteri	-	-	-	-	0.13	0.91	**0.033**	0.19
Colon & rectum	1.51	2.58	0.14	0.59	2.14	2.08	0.93	1.03
Corpus uteri	-	-	-	-	3.40	1.95	0.08	1.75
Esophagus	0.13	0.26	0.60	0.58	0.13	0.00	0.48	2.92
M. Hodgkin	0.13	0.00	0.48	2.93	0.13	0.00	0.48	2.92
Kidney & renal pelvis	0.13	0.52	0.20	0.32	0.13	0.26	0.59	0.58
Larynx	0.50	0.52	0.97	0.98	0.25	0.00	0.24	4.87
Leukemia	0.13	0.13	0.98	0.98	0.76	0.78	0.96	0.97
Liver & biliary tract	1.01	1.16	0.77	0.87	2.27	0.39	**0.0009**	5.23
Lung & bronchus	2.52	2.71	0.81	0.93	0.76	0.52	0.58	1.41
Melanoma	0.00	0.26	0.22	0.19	0.38	0.26	0.71	1.36
NHL	0.00	0.00	0.99	0.98	0.00	0.13	0.46	0.32
Oral cavity & pharynx	0.50	0.52	0.97	0.98	0.00	0.00	0.99	0.98
Ovary	-	-	-	-	0.38	0.26	0.71	1.36
Pancreas	0.88	0.13	**0.039**	4.92	0.25	1.04	0.056	0.28
Prostate	1.39	2.97	**0.032**	0.47	-	-	-	-
Stomach	3.15	1.94	0.13	1.63	2.14	0.52	**0.005**	3.84
Testis	0.38	0.26	0.70	1.37	-	-	-	-
Thyroid	0.00	0.13	0.46	0.32	0.25	0.00	0.24	4.87
Urinary bladder	0.13	0.52	0.20	0.32	0.25	0.00	0.24	4.87

p-values < 0.05 are typed in bold letters; in order to estimate odds ratios (OR) even in the presence of zero counts the value of 0.5 was added to all counts.

We found a significantly increased cancer history (OR 0.19; 95% CI 0.02 to 0.88; p = 0.033) for cancer of cervix uteri in mothers of controls (0.91%) compared with mothers of diabetic patients (0.13%). In contrast, mothers of diabetic patients showed an elevated history of cancer (OR 5.23; 95% CI 1.89 to 19.9; p = 0.0009) of the liver and biliray tract (2.27 %; 0.39% in mothers of diabetic patients and of controls, respectively). In addition, stomach cancer history was also more frequent (OR 3.84; 95% CI 1.47 to 12.4; p = 0.005) in mothers of diabetic patients (2.14%) compared to mothers of controls (0.52 %).

Cancer history was significantly elevated (OR 4.92; 95% CI 1.07 to 46.7; p = 0.039) in fathers of diabetic patients concerning pancreatic cancer (0.88% compared with 0.13% in fathers of controls). Most notably, the paternal prostate cancer history was decreased (OR 0.47; 95% CI 0.22 to 0.94; p = 0.032) among fathers of diabetic patients. 1.39% of the fathers of the cases were reported to suffer from prostate cancer, in contrast to 2.97% of the fathers of the controls. There was no statistically significant difference between the histories of other malignancies in both groups.

Subsequently, the enlarged study included a total sample of 801 type 2 diabetes patients and 1267 controls. Statistically significant differencies in cancer histories, which are presented in Table 2, were found in cancers of stomach and prostate.

**Table 2:** Frequencies of cancers in parents of 801 diabetic patients and 1267 controls

	**Fathers of**				**Mothers of**			
**Type of cancer**	**diabetic patients**	**controls**			**diabetic patients**	**controls**		

	**%**	**%**	**p**	**OR**	**%**	**%**	**p**	**OR**

Basalioma	0.00	0.00	0.82	1.58	0.12	0.00	0.30	4.75
Breast	0.00	0.08	0.68	0.53	3.62	3.95	0.73	0.92
Colon & rectum	1.50	2.05	0.38	0.74	2.12	2.13	0.98	1.01
Leukemia	0.12	0.39	0.33	0.43	0.75	0.55	0.56	1.37
Lung & bronchus	2.50	2.53	0.99	1.00	0.75	0.71	0.88	1.08
Melanoma	0.00	0.32	0.15	0.18	0.37	0.16	0.33	2.22
Non-Hodkin-Lymphoma	0.00	0.00	0.82	1.58	0.00	0.16	0.40	0.32
Prostate	1.37	2.84	**0.03**	0.49	-	-	-	-
Stomach	3.12	1.50	**0.01**	2.10	2.12	0.79	**0.01**	2.67

p-values < 0.05 are typed in bold letters; in order to estimate odds ratios (OR) even in the presence of zero counts the value of 0.5 was added to all counts.

Stomach cancer was significantly higher among mothers of controls (OR 2.67; p = 0.0106) and among fathers of diabetic patients (OR 2.10; p = 0.0141). Interestingly, prostate cancer in fathers of type 2 diabetic patients showed again a lower number than in fathers of controls (OR 0.49; p = 0.0279).

However, when the Mantel-Haenszel statistics was applied, no statistically significant results in differences between cancer histories were observed (Table 3).

**Table 3:** Frequencies of cancers in parents of 801 diabetic patients and 1267 controls (Mantel-Haenszel statistics)

	**Fathers of**			**Mothers of**		
**Type of cancer**	**diabetic patients**	**controls**		**diabetic patients**	**controls**	

	**%**	**%**	**OR**	**%**	**%**	**OR**

Basalioma	0.00	0.00	1.71	0.12	0.00	1.92
Breast	0.00	0.08	1.48	3.62	3.95	0.95
Colon & rectum	1.50	2.05	0.81	2.12	2.13	0.95
Leukemia	0.12	0.39	0.87	0.75	0.55	1.24
Lung & bronchus	2.50	2.53	0.88	0.75	0.71	0.81
Melanoma	0.00	0.32	0.94	0.37	0.16	1.81
Non-Hodkin-Lymphoma	0.00	0.00	1.71	0.00	0.16	1.30
Prostate	1.37	2.84	0.61	-	-	-
Stomach	3.12	1.50	1.77	2.12	0.79	1.68

## Discussion

The main findings in this case-control study conducted in Southern Germany demonstrate that prostate cancer is reduced in fathers of type 2 diabetic patients. In addition, an elevated history of cancer of cervix uteri was found in mothers of controls compared with mothers of diabetic patients. Furthermore, the history of cancers of the liver, biliary tract and stomach was increased in mothers of cases. Besides, we showed that the history of pancreatic cancer was higher in fathers of diabetic patients. On the basis of epidemiological and biological data we had selected prostate cancer as the main point of investigation in our study. The observed associations of other types of cancers with diabetes are presented as secondary results. Therefore, an adjustment of p-values for multiple testing was not done.

A reduced prostate cancer incidence in patients suffering from type 2 diabetes was found in various other studies [4,7,15] presuming the existence of a diabetes protective effect on prostate cancer. Our data also demonstrate that fathers of diabetic patients show a lower number of prostate cancers compared to fathers of healthy controls. This lower history of prostate cancer in fathers of diabetic patients can be connected with common ways of nutrition as well as shared genetic factors since first grade relatives have 50% of their genes in common. Thus, our findings confirm the hypothesis of a diabetes protective effect on prostate cancer and emphasise the important role of genetic factors in this progress. Since the median age of the case group was substantial higher than the median age of the controls, fathers of diabetic patients even had a higher possibility of developing prostate cancer as its incidence rises by age. Therefore, the observed reduction of paternal prostate cancer is unlikely to be due to bias on the different median ages of the case and control groups.

This protective role of diabetes on prostate cancer may be explained in terms of decreased levels of insulin-like growth factor I (IGF-I) which have been reported in diabetic patients with hyperinsulinemia by some studies [16-19] In addition, chronic hyperinsulinemia was found to be inversely associated with plasma testosterone levels [20]. Clinical and epidemiological observations suggest that both IGF-I and androgens may enhance prostate tumour development [20-22]. In view of the alterations in serum testosterone and IGF-I concentrations caused by diabetes mellitus, a reduction of prostate cancer risk among humans with a genetic background of diabetes appears biologically plausible [16].

Another possible explanation for the diabetes protective effect on prostate cancer could be the existence of a genetic factor that on one hand raises the risk of diabetes while on the other hand lowering the prostate cancer risk. Genetic variation in *PPAR-gamma *(MIM# 601487) e.g. was found to be associated with a higher incidence of diabetes mellitus [23,24] In addition, PPAR-gamma was shown to be expressed in human prostate adenocarcinomas and derived cell lines. Moreover, the activation of this receptor with specific ligands leads to an inhibition of prostate cancer cell growth [25].

In conclusion, we report for the first time that fathers of type 2 diabetic patients show decreased history of prostate cancer. A protective effect of diabetes predisposing genetic factors on prostate cancer genesis may be assumed.

## Abbreviations

CI (confidence interval)

HbA1c (glycosylated hemoglobine)

IGF-1 (insulin-like growth factor I)

OR (odds ratio)

PPAR-gamma (peroxisome proliferator-activated receptor-gamma)
